# Status and Influencing Factors of Social Media Addiction in Chinese Workers: Cross-Sectional Survey Study

**DOI:** 10.2196/48026

**Published:** 2024-03-06

**Authors:** Weitao Kong, Yuanyuan Li, Aijing Luo, Wenzhao Xie

**Affiliations:** 1 The Second Xiangya Hospital Central South University Changsha China; 2 School of Life Sciences Central South University Changsha China; 3 Key Laboratory of Medical Information Research Central South University, College of Hunan Province Changsha China; 4 Clinical Research Center For Cardiovascular Intelligent Healthcare In Hunan Province Changsha China; 5 Department of Geratology Hunan Provincial People’s Hospital The First Affiliated Hospital of Hunan Normal University Changsha China; 6 The Third Xiangya Hospital Central South University Changsha China

**Keywords:** social media addiction, job burnout, mindfulness, mobile phone, technology addiction, cross-sectional survey

## Abstract

**Background:**

Social media addiction (SMA) caused by excessive dependence on social media is becoming a global problem. At present, most of the SMA studies recruit college students as research participants, with very few studies involving workers and other age groups, especially in China.

**Objective:**

This study aims to investigate the current status of SMA among Chinese workers and analyze its influencing factors.

**Methods:**

From November 1, 2022, to January 30, 2023, we conducted an anonymous web-based questionnaire survey in mainland China, and a total of 5176 participants completed the questionnaire. The questionnaire included the Social Networking Service Addiction Scale, Maslach Burnout Inventory–General Survey scale, Mindful Attention Awareness Scale, as well as questionnaires regarding participants’ social media use habits and demographic information.

**Results:**

Through strict screening, 3468 valid questionnaires were included in this study. The main findings of this study revealed the following: the average SMA score of workers was higher (mean 53.19, SD 12.04), and some of them (393/3468, 11.33%) relied heavily on social media; there were statistically significant differences in SMA scores among workers in different industries (*F*_14,3453_=3.98; *P*<.001); single workers (*t*_3106_=8.6; *P*<.001) and workers in a relationship (*t*_2749_=5.67; *P*<.001) had higher SMA scores than married workers, but some married workers (214/3468, 6.17%) were highly dependent on social media; the level of SMA among female workers was higher than that of male workers (*t*_3466_=3.65; *P*<.001), and the SMA score of workers negatively correlated with age (*r*=−0.22; *P*<.001) and positively correlated with education level (*r*=0.12; *P*<.001); the frequency of using social media for entertainment during work (*r*=0.33; *P*<.001) and the frequency of staying up late using social media (*r*=0.14; *P*<.001) were positively correlated with the level of SMA in workers; and the level of SMA in workers was significantly positively correlated with their level of burnout (*r*=0.35; *P*<.001), whereas it was significantly negatively correlated with their level of mindfulness (*r*=−0.55; *P*<.001).

**Conclusions:**

The results of this study suggest that SMA among Chinese workers is relatively serious and that the SMA problem among workers requires more attention from society and academia. In particular, female workers, young workers, unmarried workers, highly educated workers, workers with bad social media habits, workers with high levels of job burnout, and workers with low levels of mindfulness were highly dependent on social media. In addition, occupation is an important influencing factor in SMA. Thus, the government should strengthen the supervision of social media companies. Medical institutions should provide health education on SMA and offer intervention programs for those addicted to social media. Workers should cultivate healthy habits while using social media.

## Introduction

### Background

In 2022, there were 4.62 billion active social media users worldwide, and each user was reported to spend an average of 2.5 hours a day on social media [1]. On the one hand, social media improves the efficiency of people’s communication and work and provides new ways of entertainment. On the other hand, many scholars have pointed out that poor use of social media has become a new addiction [2]. Social media addiction (SMA) is a type of technology addiction that manifests as excessive attention to social media, strong motivation to use social media, and a large amount of energy expended toward the use of social media, which may affect physical health, mental health, interpersonal relationships, study and work efficiency, and happiness in life [3].

SMA is becoming a global problem, and research in many countries has found that some residents are overly dependent on social media [4]. From the perspective of age distribution, teenagers [5], middle-aged people [6], and some older adults [7] may have excessive dependence on social media. Previous studies have found that SMA has many adverse effects on people’s physical and mental health. In terms of physical health, SMA is associated with poor eating habits of adolescents [8], making them addicted to food [9] or anorexia [10], which causes more adverse physiological reactions [11], and SMA can also reduce the quality of sleep in young people [12], which affects their executive function [13]. SMA has a negative impact on many aspects of mental health [14]. High levels of SMA can lead to higher levels of anxiety, depression, and stress [15]. People with high levels of SMA often feel anxious about their appearance [16], develop jealousy, and increase their willingness to undergo cosmetic surgery [17]. In addition, SMA can make students less engaged in learning, affect their academic performance [18], distract workers and lead to lower work efficiency, and make them face difficulties in career [19]. At the same time, SMA can have a negative impact on interpersonal relationships, which may lead to young romantic relationships becoming unstable [20]. People with high levels of SMA become introverted, prefer communication on the internet, ignore the importance of real social interaction, which affects their real interpersonal relationships [21].

Previous studies have identified many factors that influence SMA among young people and students. Among young people, psychological factors such as stress, narcissism, and nomophobia [22] as well as thoughts of the fear of missing out [23] have been found to increase their dependence on social media. Studies in the adolescent population have found that family life satisfaction [24], family economic status [25], negative emotions, and peer relationships [26] are predictors of SMA. In addition, some young people’s motivations for using social media may lead to higher levels of SMA, such as escaping from reality and seeking a sense of belonging through social media or seeking social satisfaction through social media [27].

However, most previous studies were conducted among students [14]. Researchers have pointed out that SMA studies currently rely heavily on students as research participants [28]. However, little attention has been paid to the SMA of workers and middle-aged and older adults, and there are very few research data for reference. In China, in particular, there are few studies specifically on SMA among workers. Our study on physicians and nurses in China is one of the few SMA studies on Chinese workers. We found that physicians and nurses generally have high SMA levels [6]. In addition, previous research has found that some workers are addicted to the internet, which also indicates that they are at risk of technology addiction [29,30].

On the basis of the limitations of existing research, the main purpose of this study was to investigate the current status of SMA in workers (including middle-aged and older adults) and analyze the influencing factors to provide theoretical support for the prevention and intervention of SMA in future.

### The Proposal of Research Hypothesis

#### Demographic Factors

Existing studies have shown that there are demographic factors, such as sex and age, that influence SMA levels; young people or women have higher levels of SMA than men and older people [31]; and there are differences in SMA levels based on marital status and income satisfaction [6]. Educational level is also a potential influencing factor [7]. Unreasonable social media use also increases the risk of SMA [32]. In existing studies, the definition of the unreasonable use of social media is not uniform [33]. We used the definition of SMA by Andreassen and Pallesen [3], which is recognized by many researchers in academia, that is, whether there will be adverse consequences, to judge whether the use behavior is reasonable. Considering the characteristics of workers’ lives, their unreasonable use of social media is mainly reflected in their use of social media for entertainment during work and staying up late. On the basis of the abovementioned findings, we propose hypothesis 1 and hypothesis 2:

- H1: The SMA of Chinese workers is related to demographic factors.
- H2: The SMA of workers is positively correlated with the frequency of using social media for entertainment while working and the frequency of staying up late to use social media.

#### Burnout

Burnout is a prolonged response to chronic emotional and interpersonal stressors on the job [34]. It manifests as an imbalance between effort put into work and the results achieved [35] or as a response to work stress and work overload [36]. Burnout is a psychosomatic syndrome characterized by emotional exhaustion, depersonalization, and reduced personal fulfillment [34]. Job burnout is common among Chinese workers, and it is a common psychological problem [37]. Our research on medical workers indicated that SMA is positively correlated with the degree of burnout [6], but there have been few studies on burnout and SMA among workers in other industries. However, it remains to be seen whether this relationship can be observed among the entire workforce. On the basis of the current evidence, we propose the following third hypothesis:

H3: The SMA level of Chinese workers is positively correlated with the level of job burnout.

#### Mindfulness

Derived from Eastern Buddhist meditation, mindfulness is defined as “awareness brought about by intentional, present-moment, nonjudgmental attention” [38]. Mindfulness can be studied as an intervention method, effect, or trait [39]. Meta-analysis studies have shown that mindfulness interventions are significant in improving physical and mental symptoms and are effective in relieving physical symptoms [40] as well as psychological symptoms [41]. Therefore, mindfulness training has been widely applied to combat addictive behaviors. At present, studies have confirmed a strong negative correlation between mindfulness and technology addiction. For example, a lack of mindfulness can aggravate smartphone addiction in adults, children, and adolescents [42,43], and people with low mindfulness are usually more prone to internet addiction [41]. At present, there are few research data on mindfulness and SMA, and the relationship between mindfulness and SMA in workers remains to be studied. On the basis of the correlation between mindfulness and addictive behaviors, we propose hypothesis 4:

H4: The level of SMA in Chinese workers is negatively correlated with the level of mindfulness.

## Methods

### Study Design and Procedure

We conducted an anonymous web-based questionnaire survey among Chinese workers to investigate their SMA status and influencing factors. The total questionnaire included the Social Networking Service Addiction Scale (SNSAS), the Maslach Burnout Inventory–General Survey (MBI-GS) scale, the Mindful Attention Awareness Scale (MAAS), a demographic data questionnaire, and a social media use habit questionnaire. We used WJX [44] to create the questionnaire as well as the link and QR code for the questionnaire. WJX is widely used in questionnaire research in China [6], and it is efficient and reliable.

### Recruitment

We used random sampling methods to select 2 provinces each from eastern, central, and western China, namely Guangdong, Jiangsu, Hunan, Hubei, Sichuan, and Yunnan. Our research team searched the official websites of the 6 provincial trade unions, obtained their contact information, and contacted them for data collection support. After obtaining the consent of the 6 trade unions, the trade union staff distributed our questionnaire to various work-related group chats on WeChat in the region. They also encouraged participants who completed the questionnaire to further share it within their social media networks. We also publicly released the questionnaire on the official website of the Key Laboratory of Medical Information Research of Central South University, and any participant interested in participating in the survey could fill out the questionnaire through the link. Through the abovementioned efforts, our questionnaire has been widely used on Chinese social media. The formal questionnaire survey was completed from November 1, 2022, to January 30, 2023. On the basis of the principle of voluntary participation, a total of 5176 people ultimately completed the survey questionnaire. After screening, 3468 valid questionnaires were received.

In our questionnaire survey system, we had set all questions to be completed before submitting the questionnaire. Therefore, all submitted questionnaires were complete. The following methods, including specific inclusion and exclusion criteria as well as screening criteria for invalid questionnaires, were used to ensure the quality of the questionnaire in this study. The inclusion criteria were as follows: (1) participants must have a full-time job or be self-employed (participants were asked to fill in their own occupation names), (2) participants who choose “yes” on the question “Do you understand all the questionnaire questions,” and (3) participants who choose “yes” on the question of “Are you an adult?” The exclusion criteria were as follows: (1) participants who filled in “student,” “retired,” or “unemployed” for the question “What is your occupation?” and (2) participants who answered “no” to the question “Do you work full-time?” The screening criteria for invalid questionnaires were as follows: (1) participants who completed the questionnaire in <300 seconds (lower than the normal answering time) and (2) participants who answered polygraph questions incorrectly.

### Measures

#### SNSAS Scale

We measured participants’ SMA levels using a scale developed by Chinese researchers [45] based on the Bergen Facebook Addiction Scale [46]. This scale has been successfully applied to workers in mainland China [6]. This scale has 6 dimensions: mood modification, salience, tolerance, withdrawal, conflict, and relapse. The SNSAS has 18 questions with a total score of 90 points, with higher scores indicating a more serious SMA. Each question is assessed on a 5-point Likert scale. Cronbach α coefficient of the scale in this study was 0.92.

#### MBI-GS Scale

The MBI-GS is a revised version of the MBI scale. It is a widely used tool to measure burnout among different working groups in China [47]. The Chinese version of the MBI-GS scale consists of 15 questions divided into 3 dimensions: emotional exhaustion with 5 questions, depersonalization with 4 questions, and personal accomplishment with 6 questions [48]. Each item was evaluated using a 7-point Likert scale. The higher the score, the higher the level of burnout. In this study, Cronbach α coefficient of the MBI-GS was 0.88.

#### MAAS Scale

This study used the Chinese version of the MAAS to measure participants’ mindfulness levels [49]. MAAS is a common tool used to collect mindfulness data in China [49]. The Chinese version of the MAAS is a 1D scale consisting of 15 questions scored on a 6-point Likert scale, with a higher score indicating a higher level of mindfulness. Cronbach α coefficient of the MAAS in this study was 0.92.

#### Demographic Variables and Other Variables

We also identified the following 6 demographic variables that may have affected the data through literature analysis and expert consultation: work industry, sex, age, marital status, monthly income, and educational background. We measured respondents’ bad social media habits using a single-item question, for example, “How many days a week do you stay up late using social media?” All the questions were scored on a 4 to 7 Likert scale.

### Statistical Analysis

SPSS software (version 24.0; IBM Corp) was used for data analysis. The differences in SMA levels between participants of different sex and marital status were analyzed using a 2-tailed *t* test. ANOVA was used to examine the relationship between SMA scores and discontinuous variables such as marital status. Pearson correlation coefficient was used to measure the correlation between continuous variables such as SMA, burnout, and mindfulness, and linear regression analysis was used to test the effect of independent variables on SMA outcomes. Statistical significance was set at *P*<.05.

### Ethical Considerations

All research procedures were approved by the Ethics Committee of the School of Life Sciences, Central South University (Number 2022-1-46). Before the survey, each participant was given an web-based informed consent form specifying the purpose of this study. The survey was conducted anonymously.

## Results

### Sociodemographic Characteristics and Social Media Use Habits of the Participants

Table 1 lists all social demographic characteristics and the corresponding means and SDs of the SMA scores. We used International Standard Industry Classification 4.0, and the occupations of all participants were manually categorized into 15 categories; the largest number of workers was in the education industry, which was dominated by teachers and educational ancillary occupations (992/3468, 28.6%). The lowest number of workers was in the transportation and storage industry, which mainly included workers in the passenger transport industry and logistics industry. The proportion of workers in the other 13 industries ranged from 2.02% (70/3468) to 10.78% (374/3468) of the total number industries that were inconvenient to classify, such as the funeral industry, were classified as “other service activities,” accounting for 4.64% (161/3468) of the total number of workers. Of the 3468 workers, 2029 (58.51%) were women. The participants’ age ranged from 18 to 69 years, with 31.6% (1096/3468) of the middle-aged workers aged 36 to 45 years. Moreover, 39.73% (1378/3468) of the workers were aged between 18 and 35 years, and workers aged >56 years accounted for only 1.99% (69/3468) of the total workforce sample. Overall, 68.95% (2391/3468) of the participants were married, 20.67% (717/3468) of the participants were single, and 10.38% (360/3468) of the participants were in a relationship. The monthly income of most participants (2736/3468, 78.89%) ranged from RMB 2000 to RMB 10,000 (a currency exchange rate of RMB ¥1=US $0.15 is applicable), with 2.62% (91/3468) of the participants in the highest income group (>RMB 20,000) and 3.2% (111/3468) of the participants in the lowest income group (<RMB 1000). Most of the participants (1527/3468, 44.03%) had a bachelor’s degree. Table 2 lists the frequency of undesirable social media use by all participants. Overall, 6.55% (227/3468) of the workers used social media in inopportune situations such as work and meetings, 43.63% (1513/3468) of the workers used it whenever they were free, 42.47% (1473/3468) of the workers used it when they were bored, and only 7.35% (255/3468) rarely used it. Of the 3468 participants, 3374 (97.29%) reported staying up late using social media at least 1 day per week and 849 (24.48%) reported staying up late using social media every day. Only 2.71% (94/3468) of the participants said they never stayed up late using social media.

**Table 1:** Sociodemographic characteristics and Social Networking Service Addiction Scale (SNSAS) score of the participants (N=3468).

Characteristics			Values, n (%)		SNSAS score, mean (SD)
SNSAS scores of all participants			3468 (100)		53.19 (12.04)
**Occupation**					
	Agriculture, forestry, and fishing	289 (8.33)		50.43 (11.44)	
	Manufacturing	176 (5.07)		51.15 (11.74)	
	Construction	374 (10.78)		52.40 (11.60)	
	Wholesale and retail trade; repair of motor vehicles and motorcycles	263 (7.58)		52.41 (11.96)	
	Transportation and storage	52 (1.5)		51.27 (11.43)	
	Accommodation and food service activities	70 (2.02)		53.91 (12.01)	
	Information and communication	83 (2.39)		50.41 (11.19)	
	Financial and insurance activities	117 (3.37)		53.74 (10.46)	
	Professional, scientific, and technical activities	135 (3.89)		53.84 (12.10)	
	Administrative and support service activities	190 (5.48)		53.72 (11.97)	
	Public administration and defense; compulsory social security	200 (5.77)		54.28 (12.84)	
	Education	992 (28.6)		54.81 (12.04)	
	Human health and social work activities	99 (2.85)		54.67 (12.71)	
	Activities of households as employers; undifferentiated goods- and services-producing activities of households for own use	267 (7.7)		53.25 (12.40)	
	Other service activities	161 (4.64)		51.31 (12.59)	
**Sex**					
	Male	1439 (41.49)		52.31 (12.17)	
	Female	2029 (58.51)		53.82 (11.91)	
**Age (y)**					
	18-25	755 (21.77)		56.91 (11.49)	
	26-35	623 (17.96)		55.64 (11.86)	
	36-45	1096 (31.6)		52.08 (12.02)	
	46-55	925 (26.67)		50.07 (11.58)	
	56-69	69 (1.99)		49.91 (10.89)	
**Marital status**					
	Single (include divorced or widowed)	717 (20.67)		56.26 (12.27)	
	In a relationship	360 (10.38)		55.67 (11.44)	
	Married	2391 (68.95)		51.90 (11.83)	
**Monthly income (RMB ¥^a^)**					
	500-1000	111 (3.2)		52.17 (12.37)	
	1000-1500	101 (2.91)		52.87 (11.94)	
	1500-2000	161 (4.64)		51.76 (13.07)	
	2000-3000	547 (15.77)		53.24 (11.48)	
	3000-5000	1167 (33.65)		53.93 (11.86)	
	5000-10,000	1022 (29.47)		53.07 (12.29)	
	10,000-20,000	268 (7.73)		52.52 (11.76)	
	>20,000	91 (2.62)		50.80 (12.83)	
**Educational background**					
	No education	27 (0.78)		51.04 (12.55)	
	Primary school	242 (6.98)		50.14 (11.34)	
	Junior high school	581 (16.75)		51.69 (11.09)	
	High school	459 (13.24)		51.57 (11.49)	
	College degree	345 (9.95)		53.15 (13.22)	
	Bachelor’s degree	1527 (44.03)		54.65 (11.97)	
	Master’s degree	248 (7.15)		54.19 (12.89)	
	Doctorate degree	39 (1.12)		51.82 (13.48)	

^a^RMB ¥1=US $0.15.

**Table 2:** The frequency of Chinese workers using social media during work and staying up late using social media (N=3468).

Characteristics			Values, n (%)		SNSAS^a^ score, mean (SD)
**How often do you use social media for entertainment during the workday**					
	Almost all the time (even during work or meetings)	227 (6.55)		62.06 (11.99)	
	Frequent (almost all the time outside of work)	1513 (43.63)		56.05 (11.18)	
	Occasional (use when there is nothing to do)	1473 (42.47)		49.83 (10.96)	
	Hardly ever (never used unless necessary)	255 (7.35)		47.11 (13.71)	
**How many days a week do you stay up late using social media?**					
	0	94 (2.71)		46.85 (14.29)	
	1-2	286 (8.25)		44.80 (12.13)	
	3-4	1031 (29.73)		49.52 (10.56)	
	5-6	1208 (34.83)		54.36 (10.64)	
	Every day	849 (24.48)		59.51 (11.71)	

^a^SNSAS: Social Networking Service Addiction Scale.

### Distribution of the SNSAS, MBI-GS, and MAAS Scores of the Participants

The data in Table 3 show that the SMA score of all participants was 53.19 (SD 12.04), with a median score of 54. Among the 6 SNSAS scale dimensions, the highest average score was for mood modification (mean 9.63, SD 2.4 points), followed by salience (mean 9.48, SD 2.41 points). As mentioned by other researchers, the current SMA scale can only measure the degree of SMA in participants, and there are no clear diagnostic criteria [28]. However, the average score of the participants in this study exceeded half of the total score of the scale (45 points), with 76.85% (2665/3468) of the participants scoring SNSAS >45 points and 11.33% (393/3468) of the participants scoring 67.5 points (75% of the total score), indicating that the overall SMA score of the Chinese workers was relatively high. Among the 15 industries, the highest SNSAS score was in the education industry (mean 54.81, SD 12.04), followed by those engaged in human health and social work activities (mean 54.67, SD 12.71); the lowest score was in the information and communications industry (mean 50.41, SD 11.19) and agriculture, forestry, and fishery industry (mean 50.43, SD 11.44); and the rest of the industries scored between 54.28 (12.84) and 51.15 (SD 11.74). The average burnout score of the participants was 48.34 (SD 18.24), which was lower than the MBI-GS burnout standard (score ≥50), but the SD of the data was 18.24, and the burnout scores of the participants varied significantly. More than half of the participants (1863/3468, 53.72%) had scores >50, indicating that burnout is very common among Chinese workers. The average score of the workers was 62.83 (SD 11.66). At present, there is no method to clearly classify the mindfulness score, and as the total score of the MAAS scale was just 90, we believe that Chinese workers have a high mindfulness score overall.

**Table 3:** Social Networking Service Addiction Scale (SNSAS), Maslach Burnout Inventory–General Survey (MBI-GS), and Mindful Attention Awareness Scale (MAAS) scores of the participants.

Scale		Values, mean (SD)	Values, median (IQR)
**SNSAS**		53.19 (12.04)	54 (45-61)
	Mood modification	9.63 (2.4)	10 (8-11)
	Salience	9.48 (2.41)	9 (8-11)
	Tolerance	9.21 (2.41)	9 (8-11)
	Withdrawal	8.62 (2.97)	9 (6-11)
	Conflict	8.25 (2.33)	8 (7-10)
	Relapse	8 (2.48)	8 (6-9)
**MBI-GS**		48.34 (18.24)	52 (37.33-60)
	Emotional exhaustion	12.75 (6.42)	11 (9-16)
	Depersonalization	8.25 (5.23)	8 (4-11)
	Personal accomplishment	15.25 (8.23)	17 (9-21)
MAAS		62.83 (11.66)	63 (57-71)

### The Relationship Between Demographic Variables and SMA Scores

In Table 4, we conducted a *t* test for sex, and the total SMA score of female workers was significantly higher than that of male workers (*t*_3466_=3.65; *P*<.001). ANOVA showed that there were significant differences in SMA scores among workers with different marital status (*F*_2_=46.04; *P*<.001). Furthermore, we divided marital status into 3 groups and conducted *t* test analysis for each group. The results of the data analysis showed that there was no difference in SMA scores between single and “in a relationship” workers (*t*_1075_=0.76; *P*=.45), with single workers having a higher SMA score than married workers (*t*_3106_=8.6; *P*<.001) and “in a relationship” workers having a higher SMA score than married workers (*t*_2749_=5.67; *P*<.001). The ANOVA results showed that the SMA scores were not the same for workers in different industries (*F*_14_=3.98; *P*<.001). Pearson correlation analysis showed that the age of workers was significantly negatively correlated with the SMA score (*r*=−0.22; *P*<.001). There was no correlation between monthly income level and SMA score (*r*=−0.003; *P*=.88), but educational level was significantly positively correlated with SMA score (*r*=0.12; *P*<.001). How often workers used social media unreasonably at work (*r*=0.33; *P*<.001) and stayed up late using social media (*r*=0.14; *P*<.001) was significantly positively associated with the SMA score.

**Table 4:** The results of *t* tests on the participants’ Social Networking Service Addiction Scale (SNSAS) scores and sex, and the results of analysis of variance on marital status and occupation.

Characteristic			SNSAS		Mood modification		Salience		Tolerance		Withdrawal		Conflict		Relapse
**Sex (*t* test)**															
	*t* test (*df*)	−3.65 (3466)		−7.08 (3466)		−5.48 (3466)		−2.97 (3466)		−4.03 (3466)		0.04 (3466)		2.08 (3466)	
	*P* value	<.001		<.001		<.001		.003		<.001		.96		.04	
**Marital status (ANOVA)**															
	*F* test (*df*)	46.04 (2, 3465)		66.52 (2, 3465)		78.75 (2, 3465)		30.65 (2, 3465)		31.19 (2, 3465)		9.21 (2, 3465)		3.85 (2, 3465)	
	*P* value	<.001		<.001		<.001		<.001		<.001		<.001		.02	
**Single or in a relationship (*t* test)**															
	*t* test (*df*)	0.76 (1075)		−0.53 (1075)		0.40 (1075)		1.01 (1075)		−0.14 (1075)		1.91 (1075)		1.19 (1075)	
	*P* value	.45		.60		.69		.31		.89		.06		.23	
**Single or married (*t* test)**															
	*t* test (*df*)	8.60 (3106)		9.63 (3106)		11.03 (3106)		7.20 (3106)		6.42 (1095)		4.27 (3106)		2.68 (1119)	
	*P* value	<.001		<.001		<.001		<.001		<.001		<.001		.008	
**Married or in a relationship (*t* test)**															
	*t* test (*df*)	5.67 (2749)		7.88 (3106)		7.84 (3106)		4.28 (3106)		5.34 (3106)		1.07 (3106)		0.68 (3106)	
	*P* value	<.001		<.001		<.001		<.001		<.001		.28		.50	
**Work industry (ANOVA)**															
	*F* test (*df*)	3.98 (14)		4.57 (14)		5.50 (14)		2.86 (14)		3.40 (14)		2.45 (14)		1.61 (14)	
	*P* value	<.001		<.001		<.001		<.001		<.001		.002		.07	

### The Relationship Between MBI-GS Scores, MAAS Scores, and SNSAS Scores

Pearson correlation analysis (Table 5) showed that SMA level was positively correlated with burnout level (*r*=0.35; *P*<.001) and negatively correlated with mindfulness level (*r*=−0.55; *P*<.001). Only the dimension of personal accomplishment was not correlated with salience, tolerance, and withdrawal of SMA, whereas the other dimensions were positively correlated with SNSAS score and dimension score. The mindfulness score was negatively correlated with the score of each dimension of the SNSAS. Furthermore, we used job burnout and mindfulness as independent variables and SMA as the dependent variable for the linear regression analysis (Table 6). It was found that the model passed the *F* test (*F*_2,3465_=780.36; *P*<.001). The unstandardized coefficient of job burnout was 0.07 (*P*<.001), indicating that job burnout had a significant positive influence on SMA. The unstandardized coefficient of mindfulness was −0.511 (*P*<.001), indicating that mindfulness had a significant negative effect on SMA. Comparing the standardized coefficients, the effect of mindfulness on the SNSAS score was greater than that of job burnout. The variance inflation factor values in the linear regression model were all <5, indicating no collinearity problem.

**Table 5:** The Pearson correlation analysis results between the participants’ Social Networking Service Addiction Scale (SNSAS) scores and their Maslach Burnout Inventory–General Survey (MBI-GS) scores, Mindful Attention Awareness Scale (MAAS) scores, monthly income, age, and educational background.

				SNSAS		Mood modification		Salience		Tolerance		Withdrawal		Conflict		Relapse
**MBI-GS**																
	*r*		0.35		0.31		0.30		0.27		0.25		0.27		0.29	
	*P* value		<.001		<.001		<.001		<.001		<.001		<.001		<.001	
	**Emotional exhaustion**															
		*r*	0.36		0.35		0.33		0.30		0.27		0.25		0.25	
		*P* value	<.001		<.001		<.001		<.001		<.001		<.001		<.001	
	**Depersonalization**															
		*r*	0.36		0.32		0.33		0.29		0.26		0.29		0.28	
		*P* value	<.001		<.001		<.001		<.001		<.001		<.001		<.001	
	**Personal accomplishment**															
		*r*	0.06		0.03		0.03		0.03		0.03		0.07		0.11	
		*P* value	<.001		.04		.08		.12		.07		<.001		<.001	
**MAAS**																
	*r*		−0.55		−0.43		−0.45		−0.44		−0.40		−0.46		−0.47	
	*P* value		<.001		<.001		<.001		<.001		<.001		<.001		<.001	
**Monthly income**																
	*r*		−0.003		−0.04		0.01		0.00		0.01		0.03		−0.02	
	*P* value		.88		.02		.69		.83		.50		.11		.16	
**Age**																
	*r*		−0.22		−0.25		−0.25		−0.18		−0.19		−0.10		−0.08	
	*P* value		<.001		<.001		<.001		<.001		<.001		<.001		<.001	
**Educational background**																
	*r*		0.12		0.09		0.16		0.09		0.12		0.08		0.02	
	*P* value		<.001		<.001		<.001		<.001		<.001		<.001		.31	

**Table 6:** Linear regression analysis results of Social Networking Service Addiction Scale (SNSAS) scores, Maslach Burnout Inventory–General Survey (MBI-GS) scores, and Mindful Attention Awareness Scale (MAAS) scores for all participants (dependent variable: SNSAS scores).

	Unstandardized coefficients value	Standardized coefficients value	*P* value	95% CI	VIF^a^
MBI-GS score	0.07	0.11	<.001	0.05 to 0.10	1.29
MAAS score	−0.51	−0.50	<.001	−0.54 to −0.48	1.29

^a^VIF: variance inflation factor.

## Discussion

### Principal Findings

Our study is one of the largest SMA studies conducted among workers. This study investigated the status quo of SMA among Chinese workers and analyzed its influencing factors. The results showed that the average level of SMA among Chinese workers was relatively high, and some workers had serious dependence on social media. Moreover, the results of data analysis also confirm our hypotheses 1 (except for “income”), 2, 3, and 4. The level of SMA among workers varies significantly across many demographic items and is positively correlated with the frequency of negative use of social media. At the same time, the level of SMA among workers was also positively affected by job burnout and negatively affected by mindfulness.

### Demographic Differences in SMA Levels Among Workers

#### Occupation

To the best of our knowledge, this study is the first to simultaneously collect data on the level of SMA among multiple professional workers and compare their SMA levels. Previous studies have pointed out that the lack of cross-comparison owing to differences in sampling and classification is one of the shortcomings of current research on SMA [50]. A study reviewed SMA research in 32 countries and found that different studies used different SMA measurement tools, and different studies had different standards for grading SMA levels [51]. Therefore, this study makes up for the shortcomings of the existing research. Statistically significant differences in SMA levels among workers in different occupations are one of the important findings of this study. Future research on SMA should consider the impact of occupation on the SMA levels of study participants.

#### Marital Status

The data showed that unmarried people had higher SMA scores than married people. Previous studies have also shown that unmarried people have a higher risk of SMA than married people [52]. Therefore, we conducted a deeper study on the relationship between marital relationships and personal SMA levels. First, it is worth noting that the data we collected this time showed that there were many married workers with high SMA scores, which accounted for 54.45% (214/3468) of all participants with scores >67.5. This means that although the average SMA score among married groups was not high, they still had a risk of SMA. Previous studies have found that family disharmony may lead to SMA among family members [53]. If married workers have a weak marital relationship, they may need social media to meet their communication and entertainment needs. In addition, this is the first SMA study to divide participants’ marital status into 3 categories, namely, “single,” “in a relationship,” and “married.” The results showed that although both love-involved and married workers had their own partners, there was a significant statistical difference in their SMA scores. Workers who are in love may need social media more to stay in touch, and attachment anxiety is a common factor that causes SMA in individuals [54]. Our study further explains the relationship between marital status and SMA level. In future research, compared with whether one is married, the quality of the marital relationship and the degree of attachment anxiety are more worthy considerations as factors influencing SMA.

#### Sex, Age, and Education Level

The SMA of female workers was more serious than that of male workers, which is similar to the conclusions drawn from previous studies. Women tend to attach more importance to maintaining relationships than men, tend to use social media as a tool for interaction, and are more likely to use social media to cope with feelings of emptiness when social needs are not met, leading to higher SMA [55]. In addition, younger workers tended to have higher SMA scores, which is consistent with previous studies indicating that younger people prefer communication on the internet and entertainment [5]. In addition, the level of education of workers was positively correlated with their level of SMA. Previous studies have reached similar conclusions in mixed samples of students and workers [56]. As the education level increases, the virtual tolerance subfactor increases and the risk of SMA increases [7].

#### Monthly Income

The SMA of workers is not correlated with their monthly income, which conflicts with our research results in the medical community [6]. Some studies have shown that family income satisfaction affects the SMA of family members [25], whereas other studies have shown no correlation [57]; however, most of these studies were conducted in students who do not have a stable job to earn a stable income. It is worth mentioning that this study and our previous study in the medical care group used absolute income (monthly income) and relative income (income satisfaction) to measure the income level of workers. Existing studies have found that both absolute income and relative income can affect people’s positive or negative psychology, and the degree of impact of the 2 is quite different [58]. Therefore, in the study of worker’s SMA, the choice of income measurement standard is worthy of further study. In summary, we believe that whether there is a correlation between workers’ SMA and income is still debatable, and whether to use income satisfaction or monthly income to measure workers’ income level in SMA research also deserves further discussion.

### Workers’ SMA and Inappropriate Use of Social Media

The SMA of Chinese workers was positively correlated with the frequency of unreasonable use of social media at work and the frequency of staying up late using social media. Previous studies have found that the frequency of using social media itself increases the risk of SMA [32], and if used in inappropriate situations or at inappropriate times, such as students using social media during class or self-study, the risk is further increased [59]. For workers, unreasonable social media use is mainly manifested in using social media during work. Fear of missing out [23] may be one of the reasons why workers cannot help but use social media while at work, as they fear missing out on friends’ messages or the latest information on social media platforms. In addition, many workers with too many work tasks may only be able to squeeze in time for social media entertainment because of limited rest time.

Previous studies have shown that using social media before bedtime significantly reduces sleep efficiency [60], and poor sleep leads to burnout, which in turn leads to higher cognitive and emotional arousal, which is a core element of chronic insomnia [61]. Burnout increases the need for social media to alleviate stress for workers. Staying up late, burnout, and SMA thus form a vicious cycle.

### SMA and Job Burnout in Workers

This study found that SMA among workers was significantly positively correlated with job burnout, which is consistent with the results of our previous small-scale research [6]. In research conducted among college students, SMA is generally explained as the cause of their poor psychological state or bad habits [59]. However, in the relationship between SMA and job burnout among workers, we preferred to analyze burnout as an influencing factor. We believe that the heavy workload of Chinese workers forced them to choose social media as their main form of entertainment.

At present, the work pressure of Chinese workers is generally high, and job burnout among workers is very common [62]. For example, the main causes of job burnout among educators are overwork, concerns about students’ examination results, and insufficient social support [63]. The reason for job burnout among medical practitioners in China is that they are constantly exposed to high-risk and high-pressure work environments [64]. In addition, in the era before the popularization of social media, many Chinese workers were plagued by job burnout [65]. Therefore, SMA is definitely not the main cause of job burnout among workers. Convenience and speed are the main advantages of using social media for entertainment. It takes only a few seconds to a minute to watch a short video, and this can be done with just a smartphone [66]. Social media allows workers who have insufficient rest time to take a break during work, commuting, and even while walking or eating. Previous studies have found that using social media primarily as a means of entertainment significantly elevates an individual’s SMA level. Some studies have suggested that controlling social media use may improve workers’ productivity [67], which we believe may not be effective and may even be counterproductive in people with high burnout, depriving them of one of their few forms of entertainment and causing other adverse effects. We believe that reducing workers’ workload may be an effective way to reduce SMA among workers.

### SMA and Mindfulness in Workers

This study is one of the earliest studies to investigate the relationship between mindfulness and SMA among workers. We found a significant positive correlation between worker mindfulness and SMA. Previous studies on college students have found similar results [68]. In previous studies, mindfulness was found to be negatively correlated with a variety of addictive behaviors, including substance addiction, such as drug addiction [69], and in technology addiction, such as internet addiction [41], mindfulness has a significant restraining effect on addictive behaviors. Our findings provide further evidence of the influence of mindfulness on technology addiction and that this influence persists on SMA in workers. Meanwhile, there was a significant negative correlation between workers’ mindfulness scores and burnout scores (*r*=−0.472; *P*<.001), which may lead them to believe that they do not need to turn to social media to relieve work stress. On the basis of the findings of this study, we believe that it may be worth further research to improve workers’ level of mindfulness through mindfulness training combined with cognitive behavioral therapy to reduce their dependence on social media.

### Limitations

First, it is important to note that although this study encompasses a working population across 15 categories of occupations, many industries were not included in the sample, and the data in this paper are only from mainland China, therefore, whether the conclusions in this paper are applicable to all occupational groups or all countries or regions is uncertain. Second, the number of people in various industries collected in these data was not balanced. For example, the number of workers in the education industry was high, whereas the number of workers in the transportation and storage industry was very small. This imbalance may lead to inaccurate measurements of SMA levels in some occupational groups, and larger sample sizes are needed for research in the future. Third, in this study, social media includes most of the social media platforms commonly used by Chinese people, but we did not analyze specific social media or conduct a categorical analysis. In future research, we hope to discuss social media platforms by category or conduct empirical analysis on specific social media platforms, such as Douyin.

### Conclusions

This study investigated the current status of SMA among Chinese workers and analyzed the impact of demographic factors, social media use habits, job burnout, and mindfulness on workers’ SMA. It was found that the average level of SMA among workers was high, and it was affected by many factors. In particular, the risk of SMA among women, young people, single people, married people but whose family relationships are not harmonious, and highly educated workers deserve our attention. Many workers use social media as a main entertainment tool because of excessive work pressure and use it during work or stay up late, which makes them rely heavily on social media. Therefore, alleviating the work pressure on workers and allowing them to have more time to rest and participate in healthier activities are ways to reduce their dependence on social media. In terms of intervention strategies, the strong influence of mindfulness on workers’ SMA makes it a very valuable research direction to develop a mindfulness intervention plan for workers. Social media is an indispensable tool for workers in their daily lives and work, and every worker will inevitably use social media. The government should strengthen the supervision of social media companies as many social media platforms operate with the aim of increasing user stickiness and allowing users to spend more time on social media. Medical institutions should provide popular science on SMA to help workers understand the harm of SMA and set up intervention medical programs for SMA to help them get rid of their dependence on social media. For workers, changing their bad social media habits and choosing healthier recreational activities are effective ways to reduce the risk of SMA.

## Abbreviations

MAAS: Mindful Attention Awareness Scale
MBI-GS: Maslach Burnout Inventory–General Survey
SMA: social media addiction
SNSAS: Social Networking Service Addiction Scale

## Data Availability

The data sets generated during and analyzed during this study are available from the corresponding author on reasonable request.
